# Spinal meningeal melanocytoma: A rare meningeal tumor

**DOI:** 10.4103/0972-2327.74192

**Published:** 2010

**Authors:** Vissa Shanthi, B A. Ramakrishna, Vydehi Venkata Bheemaraju, Nandam Mohan Rao, Venkata Ramana Murthy Athota

**Affiliations:** Department of Pathology, Narayana Medical College, Nellore, Andhra Pradesh, India; 1 Department of Neurosurgery, Bollineni Superspeciality Hospital, Nellore, Andhra Pradesh, India

## Introduction

Melanocytomas are rare pigmented tumors of the central nervous system (CNS). They are benign but locally aggressive lesions and are very rarely associated with spinal localizations The term melanocytoma was first proposed in 1972 to describe a heavily pigmented foramen magnum tumor based on electron microscopic studies.[1] Clinically, the tumor occurs commonly in the fifth decade and is more common in females than males. The posterior fossa lesions mimic acoustic neuromas and meningiomas in location and radiologically. In the spine, the lesions usually present with myeloradiculopathy.

## Case History

**Figure 1:**
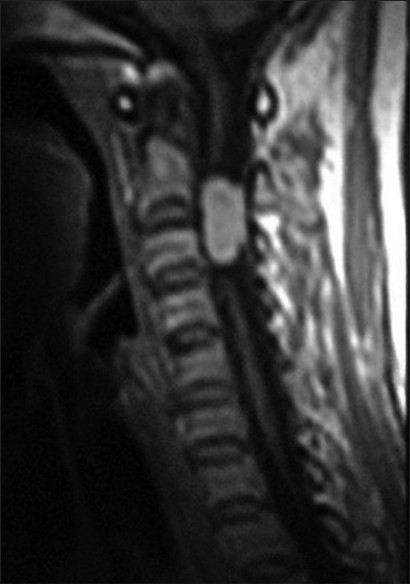
Hyperintense well-defined mass at the C2-C3 level of cervical spinal cord with wide dural attachment on T1W images of the MRI scan

**Figure 2:**
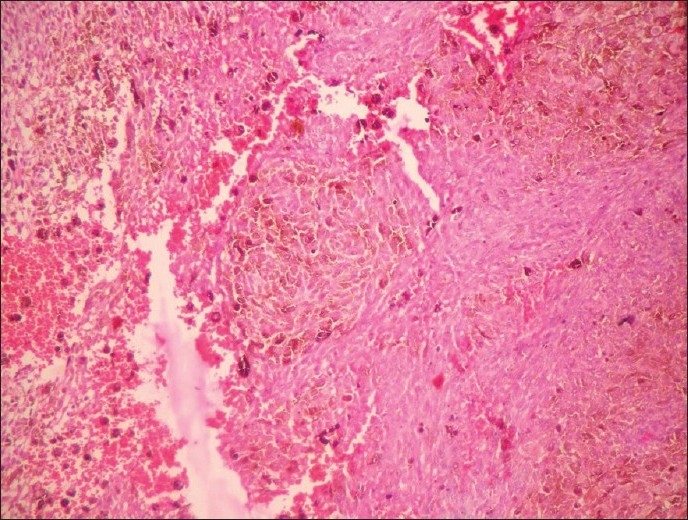
Spindle to polygonal shaped cells with intracytoplasmic pigment arranged in whorls and bundles (H and E, ×100)

**Figure 3:**
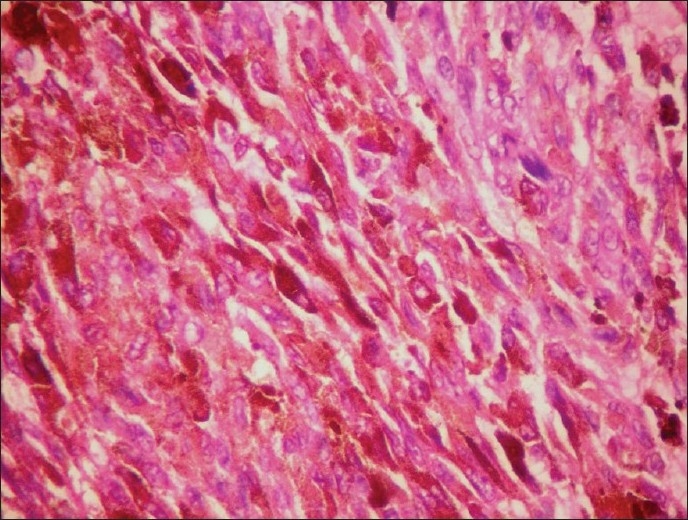
Spindle to polygonal-shaped cells with intracytoplasmic pigment and vesicular nuclei (H and E, ×400)

**Figure 4:**
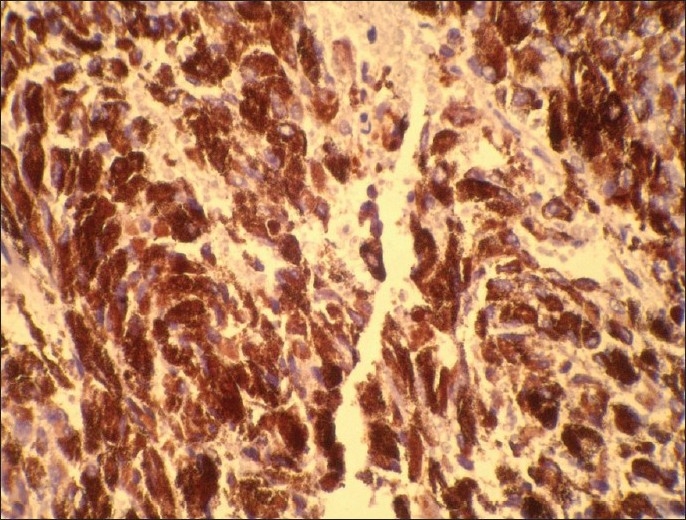
Spindle to polygonal-shaped cells with intracytoplasmic pigment showing positivity with antimelanoma antibodies (×400)

A female patient aged 30 years presented with pain in the nape of the neck extending to the left upper limb, headache, vertigo, and numbness in both upper and lower limbs. Central nervous system examination revealed tenderness over the neck region, painful movements and sensory blunting of the upper and lower limbs with signs of myelopathy.. The MRI scan showed a hyperintense well-defined mass at C2–C3 level of cervical spinal cord with wide dural attachment on T1W images [Figure 1]. Intraoperatively, dura was discolored black and an oval shaped blackish brown mass measuring 2.5×1.5×1.5cms with broad dural attachment on the left side was seen compressing the spinal cord severely. The mass was separated and radical dural excision was done and sent for histopathologic examination, which revealed a well circumscribed pigmented lesion composed of spindle shaped, polygonal cells arranged in bundles and whorls [Figure 2]. The cells exhibit moderate amount of eosinophilic cytoplasm, with vesicular nuclei and prominent nucleoli. There was no nuclear pleomorphism/mitosis/necrosis. Dense intracytoplasmic pigment was present in most of the cells, obscuring the cytologic details [Figure 3]. Perls Prussian blue stain was negative. On immunohistochemistry the tumor cells showed positivity with S100, Vimentin and Antimelanoma antibodies [Figure 4].

## Discussion

Melanocytomas are rare pigmented tumors of the central nervous system (CNS) that are found almost exclusively in the posterior cranial fossa and spinal cord. It develops from melanocytes normally present in the leptomeninges. They are benign but locally aggressive lesions and are very rarely associated with spinal localizations. The term melanocytoma was first proposed in 1972 to describe a heavily pigmented foramen magnum tumor based on electron microscopic studies.[1] Clinically, the tumor occurs commonly in the fifth decade and is more common in females than males. The posterior cranial fossa lesions mimic acoustic neuromas and meningiomas in location and radiologically. In the spinal canal, the lesions usually present with myeloradiculopathy.

Brat[2,3] had classified melanocytic lesions of CNS with respect to focal mass lesions as low grade (melanocytoma), intermediate grade, and high grade (melanoma). Few authors consider melanocytomas as a borderline tumor between cellular blue nevus and spindle cells melanoma.[1] The present World Health Organization classification classifies primary melanocytic lesions as diffuse melanocytosis, melanocytoma, malignant melanoma, and meningial melanomatosis[4] Of these lesions, only melanocytomas and malignant melanoma present as solitary lesions.

The differential diagnosis for solitary pigmented lesions of the CNS includes melanocytoma, pigmented schwannomas, and malignant melanomas[3,5]

Melanocytic schwannomas show compactly interwoven fascicles of pigmented spindle cells with oval nuclei. There can be mild nuclear pleomorphism with occasional mitosis[5] which was not seen in our case.

Malignant melanomas histologically consist of spindle or epithelioid cells arranged in loose nests, fasicles, or sheets with variable cytoplasmic melanin pigments. Nesting pattern is not a feature of malignant melanomas[3] Nuclear atypia and pleomorphism is seen along with numerous mitotic figures, necrosis, and microscopic invasion which were not seen in this case. Primary CNS melanomas are solitary and arise from the leptomeninges. Metastatic melanomas usually present with a primary skin lesion or history of removal of primary melanoma.[3]

In view of the absence of pleomorphism, mitosis and necrosis, a diagnosis of a spinal meningeal melanocytoma was considered.

Spinal meningeal melanocytomas may be intradural or extradural and occur from cervical to thoracolumbar region. Patient presents clinically with radiculopathy with or without myelopathy.[5]

Grossly, melanocytomas are well circumscribed, encapsulated, dark brown to black nodular tumors. Histologically, the cells are arranged in sheets, bundles, nests, and whorls surrounded by a fine network of reticulin fibers. The cells are fusiform to polygonal and epithelioid with abundant pale eosinophilic cytoplasm, and oval nucleus as seen in our case. Tight clustering of cells is also a feature of these tumors[3] Intracytoplasmic melanin pigment is present within the cells and in the histiocytes surrounding or in the vicinity of blood vessels.[7] Prominent nucleoli have also been described by a few authors[1,7] Mitotic figures are rare or absent. Necrosis and hemorrhage are also not seen.[1,5] We neither found any mitosis nor necrosis, but O’Brien[8] has reported minimal necrosis. Psammoma bodies and calcification have also been reported.[1,7]

Immunohistochemically, meningeal melanocytomas show positive cytoplasmic reactivity for S100 protein and vimentin. HMB45 and antimelanoma antibody are also strongly positive. Staining for keratin, epithelial membrane antigen (EMA), glial fibrillary acidic protein (GFAP), and neuron specific enolase (NSE) is non immunoreactive.[1,5,7]

Meningeal melanocytoma is a rare, histologically benign tumor with good prognosis when compared to melanoma. However, local aggressive behavior has been recorded, especially in cases of subtotal gross resection.[9]
